# Poly[[hexa­aqua­(μ_2_-fumarato-κ^4^ *O* ^1^,*O* ^1′^:*O* ^4^,*O* ^4′^)bis­(μ_3_-maleato-κ^4^ *O* ^1^,*O* ^1′^:*O* ^4^:*O* ^4′^)disamarium(III)] hexa­hydrate]

**DOI:** 10.1107/S1600536810045204

**Published:** 2010-11-10

**Authors:** Bao Li, Li-Xin Wu

**Affiliations:** a State Key Laboratory of Supramolecular Structure and Materials, College of Chemistry, Jilin University, Changchun 130012, People’s Republic of China

## Abstract

In the title coordination polymer, {[Sm_2_(C_4_H_2_O_4_)_3_(H_2_O)_6_]·6H_2_O}_*n*_, the Sm^III^ ion is nine-coordinated by four O atoms from three different maleate ligands, two O atoms from one fumarate ligand and three O atoms from three water mol­ecules. The fumarate ligand lies on an inversion center. Adjacent Sm^III^ ions are bridged by the maleate and fumarate ligands, forming a layer parallel to (011). The layers are further linked by inter­molecular O—H⋯O hydrogen bonds into a three-dimensional supra­molecular network.

## Related literature

For the structures of transition metal complexes with malonate ligands, see: Li *et al.* (2006 ▶); Ye *et al.* (2007 ▶); Zhu *et al.* (2007 ▶). For a related structure, see: Hansson & Thörnqwist (1975 ▶).
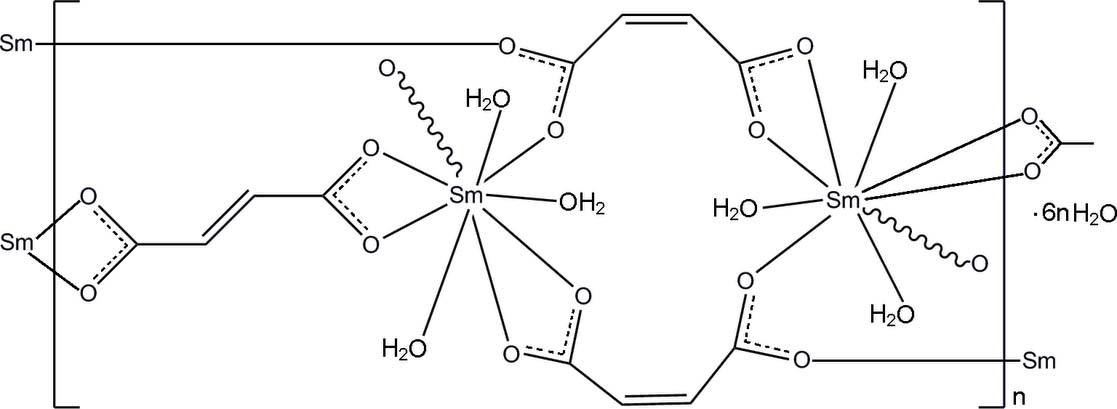


## Experimental

### 

#### Crystal data

[Sm_2_(C_4_H_2_O_4_)_3_(H_2_O)_6_]·6H_2_O
*M* *_r_* = 859.08
Triclinic, 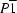

*a* = 6.150 (3) Å
*b* = 10.679 (6) Å
*c* = 11.214 (6) Å
α = 69.99 (3)°
β = 79.64 (2)°
γ = 89.74 (2)°
*V* = 679.4 (6) Å^3^
*Z* = 1
Mo *K*α radiation
μ = 4.38 mm^−1^
*T* = 290 K
0.08 × 0.07 × 0.06 mm

#### Data collection

Rigaku R-AXIS RAPID diffractometer
Absorption correction: multi-scan (*ABSCOR*; Higashi, 1995 ▶) *T*_min_ = 0.732, *T*_max_ = 0.782
6707 measured reflections
3071 independent reflections
2950 reflections with *I* > 2σ(*I*)
*R*_int_ = 0.018

#### Refinement

*R*[*F* ^2^ > 2σ(*F* ^2^)] = 0.017
*wR*(*F* ^2^) = 0.066
*S* = 1.00
3071 reflections
172 parameters
H-atom parameters constrained
Δρ_max_ = 0.58 e Å^−3^
Δρ_min_ = −0.58 e Å^−3^

### 

Data collection: *RAPID-AUTO* (Rigaku, 1998 ▶); cell refinement: *RAPID-AUTO*; data reduction: *CrystalStructure* (Rigaku/MSC, 2002 ▶); program(s) used to solve structure: *SHELXS97* (Sheldrick, 2008 ▶); program(s) used to refine structure: *SHELXL97* (Sheldrick, 2008 ▶); molecular graphics: *SHELXTL* (Sheldrick, 2008 ▶) and *DIAMOND* (Brandenburg, 1999 ▶); software used to prepare material for publication: *PLATON* (Spek, 2009 ▶).

## Supplementary Material

Crystal structure: contains datablocks global, I. DOI: 10.1107/S1600536810045204/hy2363sup1.cif

Structure factors: contains datablocks I. DOI: 10.1107/S1600536810045204/hy2363Isup2.hkl

Additional supplementary materials:  crystallographic information; 3D view; checkCIF report

## Figures and Tables

**Table 1:** Hydrogen-bond geometry (Å, °)

*D*—H⋯*A*	*D*—H	H⋯*A*	*D*⋯*A*	*D*—H⋯*A*
O7—H7*B*⋯O6^i^	0.85	1.88	2.680 (4)	156
O7—H7*A*⋯O11^ii^	0.85	1.95	2.774 (5)	164
O8—H8*A*⋯O11^iii^	0.85	1.94	2.770 (5)	164
O8—H8*B*⋯O10^ii^	0.85	1.97	2.792 (5)	164
O9—H9*A*⋯O7^iv^	0.85	2.10	2.893 (4)	155
O9—H9*B*⋯O3^iv^	0.85	1.97	2.808 (4)	168
O10—H10*A*⋯O1^v^	0.85	1.97	2.783 (4)	160
O10—H10*B*⋯O12	0.85	1.95	2.761 (5)	159
O11—H11*B*⋯O12	0.85	1.93	2.775 (5)	171
O11—H11*A*⋯O10^iv^	0.85	1.95	2.755 (5)	157
O12—H12*A*⋯O4^vi^	0.85	1.87	2.705 (5)	168
O12—H12*B*⋯O5^ii^	0.89	1.98	2.744 (5)	142

Symmetry codes: (i) 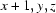; (ii) 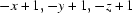; (iii) 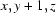; (iv) 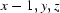; (v) 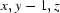; (vi) 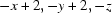.

**Fig. 1.**
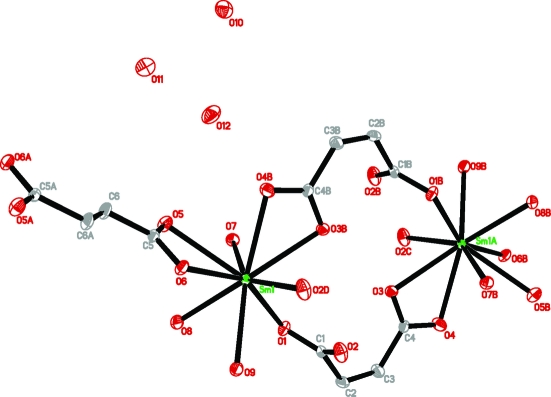
The asymmetric unit of the title compound, with the symmetry-related atoms to complete the Sm coordination. Displacement ellipsoids are drawn at the 30% probability level. H atoms have been omitted for clarity. [Symmetry codes: (A) -x, 1-y, 1-z; (B) 2-x, 2-y, -z; (C) 1+x, y, z; (D) 1-x, 2-y, -z.]

**Fig. 2.**
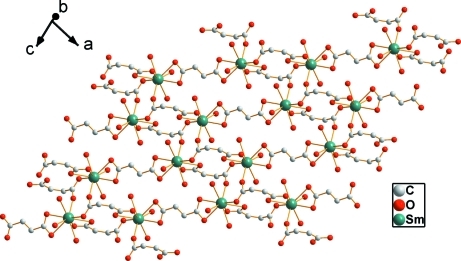
Crystal packing diagram of the title cmopound, showing the two-dimensional network.

## supplementary crystallographic information

### Comment

Diacids have been widely used to form metal–organic frameworks. Recently, we reported several compounds based on malonate ligand and different transition metal ions (Li *et al.*, 2006; Ye *et al.*, 2007; Zhu *et al.*, 2007). Hererin, we report the crystal structure of the title compound based on maleate ligand.

The structure of the title compound is shown in Fig. 1. The bond lengths and angles are normal and comparable with those reported for a similar structure (Hansson & Thörnqwist, 1975). The Sm^III^ ion is nine-coordinated by four O atoms from three maleate ligands, two O atoms from one fumarate ligand and three coordinated water molecules. The two carboxylate groups of the fumarate ligand and one of the carboxylate groups of the maleate ligand exhibit a chelating coordination mode, while the other carboxylate group of the maleate ligand binds Sm^III^ ions in a bidentate bridging mode. Adjacent Sm^III^ ions are bridged by the maleate and fumarate ligands, forming a layer parallel to (0 1 1) (Fig. 2). Additionly, abundant O—H···O hydrogen bonds stabilize the crystal structure of the title compound (Table 1).

### Experimental

Maleic acid and Sm(NO_3_)_3_ of analytical grade are used without further purification. Sm(NO_3_)_3_ (67.24 mg, 0.2 mmol) and maleic acid (69.64 mg, 0.6 mmol) were dissolved in water (10 ml), and the pH value was adjusted to about 3 using a dilute NaOH solution. The mixture was stirred for half an hour and then filtered. The filtrate was allowed to stand at room temperature for two weeks, giving colorless block-shaped crystals.

### Refinement

C-bound H atoms were positioned geometrically (C—H = 0.93 Å) and refined as riding atoms, with *U*_iso_(H) = 1.2 *U*_eq_(C). H atoms of the water molecules were initially located in a difference Fourier map, but were idealized and refined as riding atoms, with O—H = 0.85 Å and *U*_iso_(H) = 1.5*U*_eq_(O).

### Crystal data

**Table:**

[Sm_2_(C_4_H_2_O_4_)_3_(H_2_O)_6_]·6H_2_O	*Z* = 1
*M**_r_* = 859.08	*F*(000) = 418
Triclinic, *P*1	*D*_x_ = 2.100 Mg m^−^^3^
Hall symbol: -P 1	Mo *K*α radiation, λ = 0.71073 Å
*a* = 6.150 (3) Å	Cell parameters from 6497 reflections
*b* = 10.679 (6) Å	θ = 3.3–27.5°
*c* = 11.214 (6) Å	µ = 4.38 mm^−^^1^
α = 69.99 (3)°	*T* = 290 K
β = 79.64 (2)°	Block, colorless
γ = 89.74 (2)°	0.08 × 0.07 × 0.06 mm
*V* = 679.4 (6) Å^3^	

### Data collection

**Table:**

Rigaku R-AXIS RAPID diffractometer	3071 independent reflections
Radiation source: rotation anode	2950 reflections with *I* > 2σ(*I*)
graphite	*R*_int_ = 0.018
ω scans	θ_max_ = 27.5°, θ_min_ = 3.3°
Absorption correction: multi-scan (*ABSCOR*; Higashi, 1995)	*h* = −7→7
*T*_min_ = 0.732, *T*_max_ = 0.782	*k* = −13→13
6707 measured reflections	*l* = −14→13

### Refinement

**Table:**

Refinement on *F*^2^	Primary atom site location: structure-invariant direct methods
Least-squares matrix: full	Secondary atom site location: difference Fourier map
*R*[*F*^2^ > 2σ(*F*^2^)] = 0.017	Hydrogen site location: inferred from neighbouring sites
*wR*(*F*^2^) = 0.066	H-atom parameters constrained
*S* = 1.00	*w* = 1/[σ^2^(*F*_o_^2^) + (0.0342*P*)^2^ + 3.0041*P*] where *P* = (*F*_o_^2^ + 2*F*_c_^2^)/3
3071 reflections	(Δ/σ)_max_ < 0.001
172 parameters	Δρ_max_ = 0.58 e Å^−^^3^
0 restraints	Δρ_min_ = −0.58 e Å^−^^3^

### Fractional atomic coordinates and isotropic or equivalent isotropic displacement parameters (Å^2^)

**Table:**

	*x*	*y*	*z*	*U*_iso_*/*U*_eq_	
C1	0.6917 (6)	1.1675 (4)	0.0453 (3)	0.0173 (7)	
C2	0.7911 (7)	1.3067 (4)	0.0028 (4)	0.0256 (8)	
H2	0.7026	1.3681	0.0269	0.031*	
C3	0.9922 (7)	1.3525 (4)	−0.0657 (4)	0.0291 (9)	
H3	1.0311	1.4423	−0.0853	0.035*	
C4	1.1596 (6)	1.2717 (4)	−0.1134 (3)	0.0190 (7)	
C5	0.2343 (6)	0.6379 (4)	0.4136 (3)	0.0176 (7)	
C6	0.0980 (7)	0.5175 (4)	0.5083 (4)	0.0254 (8)	
H6	0.1523	0.4658	0.5807	0.031*	
O1	0.6917 (5)	1.0926 (3)	0.1584 (3)	0.0230 (6)	
O2	0.6003 (5)	1.1329 (4)	−0.0303 (3)	0.0321 (7)	
O3	1.1332 (4)	1.1467 (3)	−0.0770 (3)	0.0211 (5)	
O4	1.3316 (5)	1.3327 (3)	−0.1921 (3)	0.0326 (7)	
O5	0.4186 (5)	0.6652 (3)	0.4363 (3)	0.0253 (6)	
O6	0.1671 (4)	0.7103 (3)	0.3142 (3)	0.0224 (5)	
O7	0.8126 (4)	0.8515 (3)	0.3427 (3)	0.0223 (5)	
H7B	0.8962	0.7909	0.3325	0.033*	
H7A	0.8156	0.8534	0.4176	0.033*	
O8	0.3684 (5)	0.9438 (3)	0.4042 (3)	0.0289 (6)	
H8A	0.3148	1.0200	0.3882	0.043*	
H8B	0.3346	0.9100	0.4861	0.043*	
O9	0.1952 (4)	1.0193 (3)	0.1778 (3)	0.0236 (6)	
H9A	0.0669	0.9933	0.2236	0.035*	
H9B	0.1633	1.0477	0.1027	0.035*	
O10	0.8053 (6)	0.1975 (3)	0.3360 (3)	0.0339 (7)	
H10A	0.7981	0.1545	0.2853	0.051*	
H10B	0.7485	0.2727	0.3128	0.051*	
O11	0.2212 (5)	0.1916 (3)	0.3961 (3)	0.0322 (7)	
H11B	0.3085	0.2599	0.3771	0.048*	
H11A	0.1137	0.2035	0.3560	0.048*	
O12	0.5308 (6)	0.4066 (3)	0.3112 (3)	0.0384 (8)	
H12A	0.5696	0.4872	0.2639	0.058*	
H12B	0.5062	0.4142	0.3894	0.058*	
Sm1	0.50414 (3)	0.869449 (17)	0.223642 (16)	0.01447 (7)	

### Atomic displacement parameters (Å^2^)

**Table:**

	*U*^11^	*U*^22^	*U*^33^	*U*^12^	*U*^13^	*U*^23^
C1	0.0152 (16)	0.0201 (17)	0.0163 (16)	0.0006 (13)	0.0001 (12)	−0.0074 (13)
C2	0.028 (2)	0.0164 (17)	0.029 (2)	0.0044 (15)	0.0018 (16)	−0.0079 (15)
C3	0.035 (2)	0.0138 (17)	0.033 (2)	−0.0033 (15)	0.0089 (17)	−0.0089 (16)
C4	0.0210 (17)	0.0184 (16)	0.0152 (16)	−0.0009 (14)	−0.0014 (13)	−0.0038 (13)
C5	0.0164 (16)	0.0167 (16)	0.0163 (16)	0.0012 (13)	−0.0015 (13)	−0.0023 (13)
C6	0.0252 (19)	0.0207 (18)	0.0224 (18)	−0.0066 (15)	0.0005 (15)	0.0002 (15)
O1	0.0281 (14)	0.0196 (13)	0.0172 (12)	−0.0039 (11)	−0.0035 (11)	−0.0013 (10)
O2	0.0257 (15)	0.051 (2)	0.0247 (15)	−0.0029 (14)	−0.0051 (12)	−0.0194 (14)
O3	0.0207 (13)	0.0153 (12)	0.0247 (13)	−0.0003 (10)	−0.0003 (10)	−0.0055 (10)
O4	0.0282 (15)	0.0187 (14)	0.0409 (18)	−0.0034 (12)	0.0135 (13)	−0.0076 (13)
O5	0.0186 (13)	0.0246 (14)	0.0274 (14)	−0.0048 (11)	−0.0072 (11)	−0.0006 (11)
O6	0.0169 (12)	0.0215 (13)	0.0219 (13)	−0.0008 (10)	−0.0043 (10)	0.0017 (10)
O7	0.0154 (12)	0.0271 (14)	0.0234 (13)	0.0044 (10)	−0.0063 (10)	−0.0062 (11)
O8	0.0423 (17)	0.0262 (15)	0.0168 (13)	0.0084 (13)	−0.0019 (12)	−0.0074 (11)
O9	0.0168 (12)	0.0270 (14)	0.0216 (13)	0.0030 (11)	−0.0029 (10)	−0.0021 (11)
O10	0.0396 (18)	0.0385 (18)	0.0292 (16)	0.0048 (14)	−0.0105 (13)	−0.0168 (14)
O11	0.0342 (17)	0.0299 (16)	0.0337 (16)	0.0009 (13)	−0.0113 (13)	−0.0101 (13)
O12	0.051 (2)	0.0229 (15)	0.0373 (18)	−0.0064 (14)	−0.0083 (15)	−0.0057 (13)
Sm1	0.01201 (10)	0.01535 (10)	0.01502 (10)	−0.00140 (6)	−0.00158 (6)	−0.00444 (7)

### Geometric parameters (Å, °)

**Table:**

C1—O1	1.247 (5)	O9—H9A	0.8500
C1—O2	1.249 (5)	O9—H9B	0.8500
C1—C2	1.493 (5)	O10—H10A	0.8500
C2—C3	1.328 (6)	O10—H10B	0.8501
C2—H2	0.9300	O11—H11B	0.8500
C3—C4	1.484 (5)	O11—H11A	0.8500
C3—H3	0.9300	O12—H12A	0.8500
C4—O3	1.256 (5)	O12—H12B	0.8944
C4—O4	1.265 (5)	Sm1—O1	2.464 (3)
C5—O6	1.258 (5)	Sm1—O2^ii^	2.377 (3)
C5—O5	1.262 (5)	Sm1—O3^iii^	2.566 (3)
C5—C6	1.496 (5)	Sm1—O4^iii^	2.486 (3)
C6—C6^i^	1.327 (8)	Sm1—O5	2.593 (3)
C6—H6	0.9300	Sm1—O6	2.512 (3)
O7—H7B	0.8500	Sm1—O7	2.480 (3)
O7—H7A	0.8499	Sm1—O8	2.432 (3)
O8—H8A	0.8500	Sm1—O9	2.489 (3)
O8—H8B	0.8500		
			
O1—C1—O2	122.4 (4)	O2^ii^—Sm1—O7	145.71 (10)
O1—C1—C2	118.2 (3)	O8—Sm1—O7	73.67 (10)
O2—C1—C2	119.2 (4)	O1—Sm1—O7	71.50 (10)
C3—C2—C1	127.1 (4)	O2^ii^—Sm1—O4^iii^	76.01 (12)
C3—C2—H2	116.4	O8—Sm1—O4^iii^	137.15 (11)
C1—C2—H2	116.4	O1—Sm1—O4^iii^	126.86 (10)
C2—C3—C4	125.2 (4)	O7—Sm1—O4^iii^	80.70 (11)
C2—C3—H3	117.4	O2^ii^—Sm1—O9	71.17 (11)
C4—C3—H3	117.4	O8—Sm1—O9	69.13 (10)
O3—C4—O4	120.7 (3)	O1—Sm1—O9	77.68 (10)
O3—C4—C3	121.5 (3)	O7—Sm1—O9	136.11 (10)
O4—C4—C3	117.7 (3)	O4^iii^—Sm1—O9	143.16 (11)
O3—C4—Sm1^iii^	62.2 (2)	O2^ii^—Sm1—O6	79.19 (10)
O4—C4—Sm1^iii^	58.6 (2)	O8—Sm1—O6	84.11 (11)
C3—C4—Sm1^iii^	175.8 (3)	O1—Sm1—O6	152.27 (10)
O6—C5—O5	120.8 (3)	O7—Sm1—O6	121.53 (9)
O6—C5—C6	121.1 (3)	O4^iii^—Sm1—O6	80.77 (10)
O5—C5—C6	118.1 (3)	O9—Sm1—O6	77.11 (10)
C6^i^—C6—C5	121.8 (5)	O2^ii^—Sm1—O3^iii^	74.78 (10)
C6^i^—C6—H6	119.1	O8—Sm1—O3^iii^	140.72 (10)
C5—C6—H6	119.1	O1—Sm1—O3^iii^	76.69 (9)
C1—O1—Sm1	115.9 (2)	O7—Sm1—O3^iii^	71.03 (10)
C1—O2—Sm1^ii^	161.0 (3)	O4^iii^—Sm1—O3^iii^	51.38 (9)
C4—O3—Sm1^iii^	92.2 (2)	O9—Sm1—O3^iii^	130.71 (9)
C4—O4—Sm1^iii^	95.7 (2)	O6—Sm1—O3^iii^	129.48 (9)
C5—O5—Sm1	92.2 (2)	O2^ii^—Sm1—O5	121.78 (11)
C5—O6—Sm1	96.1 (2)	O8—Sm1—O5	70.25 (11)
Sm1—O7—H7B	110.4	O1—Sm1—O5	134.92 (9)
Sm1—O7—H7A	131.5	O7—Sm1—O5	70.75 (9)
H7B—O7—H7A	106.8	O4^iii^—Sm1—O5	69.11 (11)
Sm1—O8—H8A	118.2	O9—Sm1—O5	115.51 (9)
Sm1—O8—H8B	137.9	O6—Sm1—O5	50.82 (9)
H8A—O8—H8B	102.8	O3^iii^—Sm1—O5	112.47 (9)
Sm1—O9—H9A	118.7	O2^ii^—Sm1—C4^iii^	74.35 (11)
Sm1—O9—H9B	120.7	O8—Sm1—C4^iii^	146.16 (11)
H9A—O9—H9B	100.2	O1—Sm1—C4^iii^	101.68 (11)
H10A—O10—H10B	113.0	O7—Sm1—C4^iii^	73.74 (10)
H11B—O11—H11A	115.2	O4^iii^—Sm1—C4^iii^	25.73 (10)
H12A—O12—H12B	100.4	O9—Sm1—C4^iii^	144.26 (10)
O2^ii^—Sm1—O8	139.41 (11)	O6—Sm1—C4^iii^	105.51 (11)
O2^ii^—Sm1—O1	103.29 (11)	O3^iii^—Sm1—C4^iii^	25.66 (9)
O8—Sm1—O1	76.25 (10)	O5—Sm1—C4^iii^	90.53 (11)

Symmetry codes: (i) −*x*, −*y*+1, −*z*+1; (ii) −*x*+1, −*y*+2, −*z*; (iii) −*x*+2, −*y*+2, −*z*.

### Hydrogen-bond geometry (Å, °)

**Table:**

*D*—H···*A*	*D*—H	H···*A*	*D*···*A*	*D*—H···*A*
O7—H7B···O6^iv^	0.85	1.88	2.680 (4)	156
O7—H7A···O11^v^	0.85	1.95	2.774 (5)	164
O8—H8A···O11^vi^	0.85	1.94	2.770 (5)	164
O8—H8B···O10^v^	0.85	1.97	2.792 (5)	164
O9—H9A···O7^vii^	0.85	2.10	2.893 (4)	155
O9—H9B···O3^vii^	0.85	1.97	2.808 (4)	168
O10—H10A···O1^viii^	0.85	1.97	2.783 (4)	160
O10—H10B···O12	0.85	1.95	2.761 (5)	159
O11—H11B···O12	0.85	1.93	2.775 (5)	171
O11—H11A···O10^vii^	0.85	1.95	2.755 (5)	157
O12—H12A···O4^iii^	0.85	1.87	2.705 (5)	168
O12—H12B···O5^v^	0.89	1.98	2.744 (5)	142

Symmetry codes: (iv) *x*+1, *y*, *z*; (v) −*x*+1, −*y*+1, −*z*+1; (vi) *x*, *y*+1, *z*; (vii) *x*−1, *y*, *z*; (viii) *x*, *y*−1, *z*; (iii) −*x*+2, −*y*+2, −*z*.
